# Theoretical and Experimental Identification of Frequency Characteristics and Control Signals of a Dynamic System in the Process of Turning

**DOI:** 10.3390/ma14092260

**Published:** 2021-04-27

**Authors:** Antoni Świć, Arkadiusz Gola

**Affiliations:** Department of Production Computerisation and Robotisation, Faculty of Mechanical Engineering, Lublin University of Technology, 20-618 Lublin, Poland; a.swic@pollub.pl

**Keywords:** turning, machining, machining quality, accuracy, dynamic system characteristics, control

## Abstract

The article presents the results of the experimental validation of the developed static, time and frequency characteristics under interference and longitudinal feed control of a dynamic system in the process of turning axisymmetric parts. The experiments were conducted on a test bench, consisting of a 16B16P center lathe, a measuring system and a PC with a measurement card. The experiments were carried out to verify the assumptions of the baseline model of the turning process. As part of the study, we determined the static characteristics of the machining process, the time characteristics of the object under interference and under longitudinal feed rate control, and the frequency characteristics of the machine tool system under longitudinal feed rate control. During the experiments, we recorded the observed input and output signal curves and the observed characteristics of the interferences acting on the object, as well as the numerical values of the parameters of the equations describing the model, and in particular the gain of the elastic system, which is difficult to determine by analytical methods. The positive results of the experiments confirm the effectiveness of the proposed models and their usefulness for automation of machining processes.

## 1. Introduction

Process automation is one of the key megatrends that drive Industry 4.0 [1]. In particular, this applies to the integration and complete automation of control of production processes to the extent that decisions are made by machines, with humans playing a supervisory role [2]. As Industry 4.0 is a challenge and a goal pursued by the world’s leading economies, there is large demand for research geared towards improving existing and developing new methods and models that can assist in automating technological processes [3]. On the other hand, to ensure a high performance of a machine, it is necessary to use high quality parts. There are many factors that determine the quality of parts. They include, among others, high dimensional accuracy [4] and low surface roughness [5]. In order to produce high quality parts on an industrial scale, companies need to ensure that the production process meets high reliability standards, which can be achieved in conditions of high production automation [6]. Ensuring a high level of automation in the production of axisymmetric parts is still a serious challenge, which is why there is a need for research that is aimed at developing effective and efficient methods of machining this type of machine parts [7].

In practice, about half of all parts used in different types of machinery and mechanical devices are rotating parts [8]. They include gears, cylinders, bushings, discs, and hubs. Rotating parts, including shafts, are most commonly machined by turning. During this type of machining operation, the workpiece rotates at a certain angular velocity, which promotes vibration [9]. The vibrations that occur during machining of the shafts reduce the reliability of the turning process, negatively affecting the dimensional accuracy [10], waviness [11], and roughness of turned surfaces [12]. Therefore, the knowledge of the technical behavior of machined parts during the production process is very important—both for the technologists [13] and also for the manufacturing systems design purposes [14]. In the case of machine tools, the basic technical-and-economic criterion is machining accuracy, defined as the degree to which the actual workpiece matches the shape and dimensions of the ideal workpiece [15]. The differences between the actual and ideal workpiece are defined as errors, and the possibility of obtaining the required dimensional accuracy of a workpiece largely depends on the accuracy of the mutual spatial position of the tool and the workpiece [16]. Therefore, during a machining cycle, the required accuracy of the design motion trajectory of the tool and the workpiece should be ensured [17]. An additional reason for increasing the dimensional accuracy requirements for parts is the clear trend toward producing small batches of relatively high-cost parts in automated production lines [18].

The problem of increasing the accuracy of turning has been discussed by numerous authors. Among the solutions proposed in the literature, and the most frequently adopted is the model of interferences acting on the turning process, which consists of two components: A component representing an approximately linear trend and a random component with a wide frequency band [19]. The first component is mainly related to the temperature deformations of the machine tool and wear of the cutting edge [20]. These interferences do not change significantly within one machining cycle and can be compensated for by setting the static operating point in each subsequent machining cycle [21]. The high-frequency component is mainly conditioned by changes in machining allowance, changes in material hardness and other complex phenomena occurring during the turning process [22]. The basic high-frequency component compensation method is to control the elastic deformations of the turning process system [23].

Apart from obtaining a high machining accuracy, it is important to increase the efficiency of the turning process, which can provide a considerable economic benefit [24]. Some machine tools are constructed and operated with the aim of achieving the highest possible efficiency, while others are mainly used for precision machining [25]. Such specialization of machine tools requires the development of specific optimization and control criteria suitable for the individual classes of those tools [26]. The point of departure for controlling all classes of machine tools is the stabilization of the cutting forces and the associated elastic deformations [27].

The analysis of the literature shows that there are many publications which present solutions to particular problems with on-line machining parameters measurement and control (see e.g., [28]), stability prediction in straight turning (see e.g., [29]), or just conventional and intelligent methods for machining accuracy improvement (see e.g., [30,31]. However, there is no research devoted to the analysis of the effectiveness of advanced turning operation control methods (in particular adaptive ones) that determine static, time and frequency characteristics that describe the process in the aspect of possibility of its automation.

The goal of this article is to present the results of experimental and theoretical characteristics of turning process for various control and disruptive parameters. Moreover, implementing the design of the control system provide the possibility of increasing the accuracy of machined parts by reducing the variation of machining forces and elastic deformations was investigated.

The article comprises four sections. Section 1 presents the theoretical aspects of dynamic character of turning process, importance, challenges and necessity of developing proper control methods and models for automation of such processes and a review of relevant literature. Section 2 describes the characteristics and description of the test bench used for provided experiments. Section 3 reports the results obtained during the experimental studies and necessary discussion. In particular this section was focused on the problem of investigation of the static characteristics of the object (Section 3.1), investigation of the time characteristics of the object under interference and under longitudinal feed control (Section 3.2) and study of the frequency characteristics of the object for the feed channel (Section 3.3). The article concludes with Section 4, which contains observations and reflections made during the experiments, analyses and modelling.

## 2. Materials and Methods

**Figure 1:**
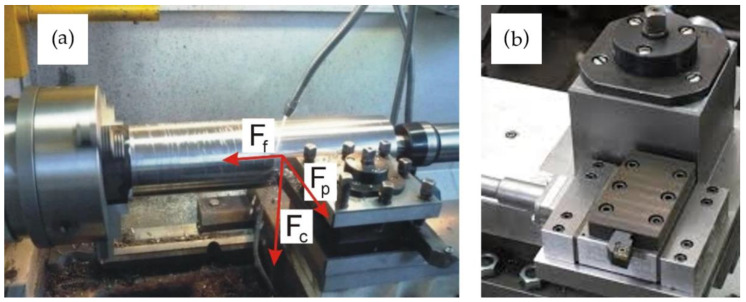
Test bench: (**a**) General view of the bench with a shaft secured in the lathe and main machining forces; (**b**) apparatus for measuring turning forces.

The test bench used in the present study was constructed on the basis of a 16″ × 40″ precise center lathe *16B16P*, produced by STANKO company (Rostov-on-Don, Russia) (Figure 1a). The tool holder was replaced with a dual-circuit strain gauge for measuring the component forces *F_c_* (tangential) and *F_f_* (axial) (Figure 1b). The results of the experiment were recorded using a PC data acquisition system integrated with a digital signal amplifier. For gathering and analysis of obtained data the HP ProBook 150 G3 computer with Intel^®^ Core™ i7-6500 CPU @ 2.5 GHz, 2592 MHz dual core processor.

The machine tool used in the tests was selected because it was in good technical condition, had sufficient spindle drive power and allowed to adjust spindle speed range and feed range to the current status of the test object.

**Figure 2:**
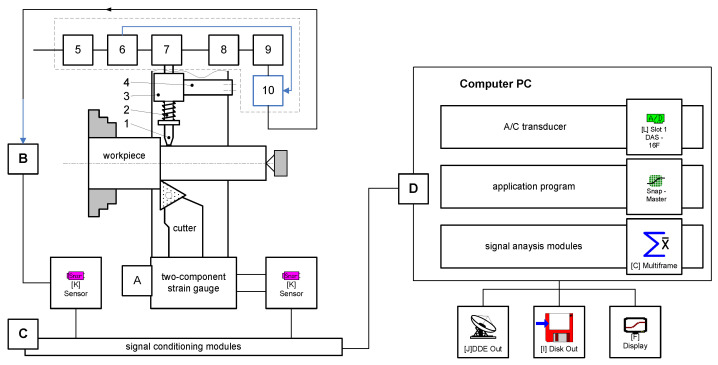
Schematic of the test bench.

The test bench (see schematic in Figure 2) consisted of two basic parts: (1) A *16B16P* center lathe and (2) a measuring system which included a PC with a measurement card.

The measuring system was comprised of:

– a strain gauge (A) for measuring the cutting force *F_c_* and the feed force *F_f_* exerted during free orthogonal turning of solids of revolution, and
– apparatus (B) for measuring elastic deformations and vibrations.

**Figure 3:**
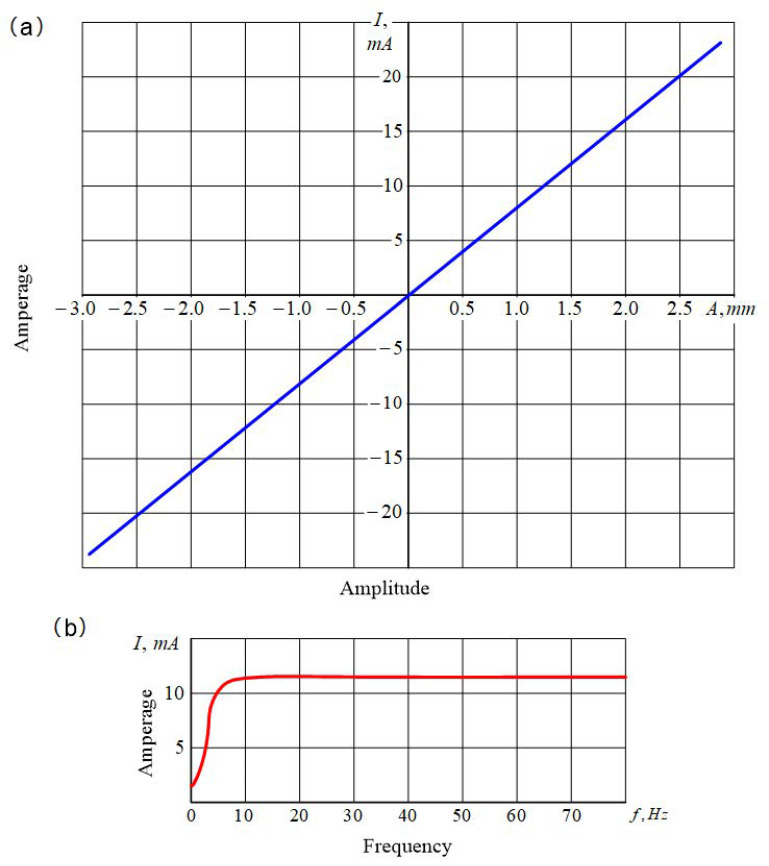
Characteristics of the elements of the block diagram: (**a**) Static characteristics of the primary transducer, (**b**) the amplitude-frequency characteristics of the primary transducer.

Elastic deformations and vibrations were measured using a *BI6-6TN* apparatus comprised of a mains voltage stabilizer (5), a voltage transducer (6), a *DW-ISG* transducer mounted on a pin 2 (7); a demodulator (8); a filter (9); and a DC amplifier (10). The static characteristic of the primary transducer 7 is shown in Figure 3a, and its amplitude-frequency characteristic is plotted in Figure 3b; the frequency response of the transducer was in the range of 10–120 Hz. Blanks were processed (Figure 1a) using a cutting edge secured in the strain gauge on the machine tool’s carriage, on which a device for measuring elastic deformations and vibrations of the workpiece was also mounted, using a bracket. The device consisted of pin 2, positioned in the ball guides of a bushing 3, and a spring 4, which had been designed to create tension.

**Figure 4:**
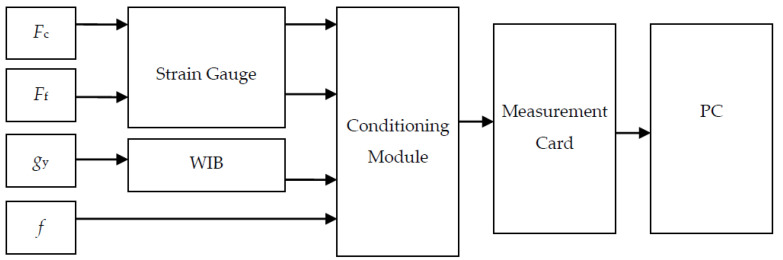
Schematic of the measurement chains.

The analog signals generated in the machining system were galvanically isolated, amplified, and filtered in the conditioning module. For this purpose, conditioning modules were used which collected signals from various sensors with a measurement signal of between 10 and 20 mV. These signals were transmitted to the PC via the measurement card. The schematic of the measurement chains has been presented in Figure 4.

The recording software stored data in text format in a hard drive file. The data could then be further processed using any program that recognized the *CSV* format, which allowed to fully control parameter registration (e.g., providing the possibility of precisely determining the moment at which the cutting edge entered the workpiece).

The developed test bench was used to analyze both static and time characteristics of the object with natural disruptions and frequency characteristics of the object for the feed channel.

## 3. Results and Discussion 

The automation of machining process requires the development of specific optimization and control criteria suitable for the individual classes of machine tools. Therefore, it is necessary to define proper models that allows to determine static, time and frequency characteristics of the object. The aim of the study was to record input and output signals and the observed characteristics of the interferences acting on the object, as well as to validate the numerical values of the parameters of the equations describing the model. For this purpose the experiments have been divided into three parts:

1. Investigations of the static characteristics of the object.
2. Investigations of the time characteristics of the object under interference and under longitudinal feed control.
3. Investigations of the frequency characteristics of the object for the feed channel.

### 3.1. Investigation of the Static Characteristics of the Object

The transition function of dynamic systems, for the control and interference channels, takes coefficients *h_ij_*, which describe the compliance of a technological system. Due to the high complexity of theoretical analysis, the values of these coefficients were determined experimentally.

To identify the static characteristics of the *16B16P* center lathe, which were later used to calculate the values of coefficients *h_ij_*, the technological system of the machine tool was subjected to a loading force simulating machining. Micrometers were used to determine the deformations of the components of the technological system for the appropriate coordinates. The load was applied using a *TG-1000* device, which generated a force acting at an angle of 60° to the vertical axis and to simulate two cutting force components: Tangential and axial or tangential and radial, depending on how the load-generating device was mounted. The experiments were carried out in accordance with the recommended methodology; before the measurements, the elastic system was repeatedly loaded with the maximum force and restored to the no-load condition.

**Figure 5:**
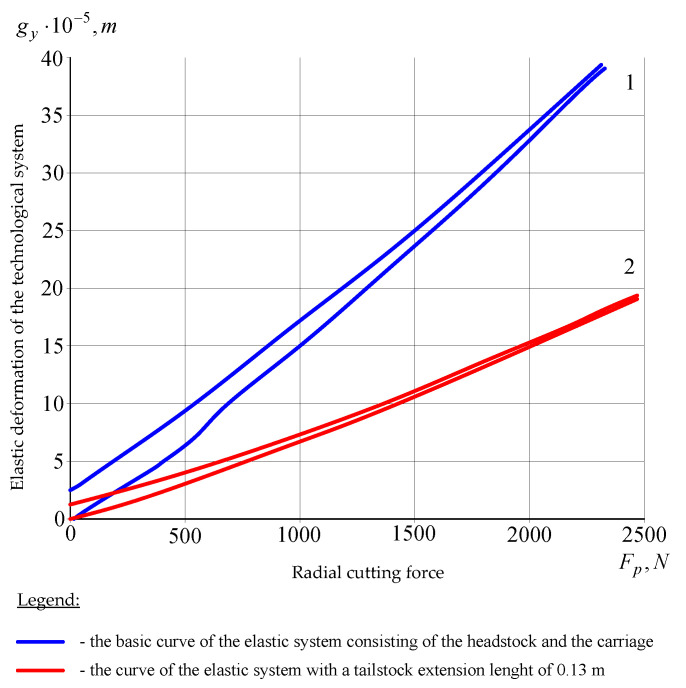
Static characteristics for the *Y* coordinate.

The *gy*(*Fp*) curve of the elastic system consisting of the headstock and the carriage is shown in Figure 5 (curve 1). The compliance of an equivalent elastic system comprising the tailstock and the carriage, as shown in the experiments, largely depended on how far the tailstock was extended. Curve 2 in Figure 5 was obtained at a tailstock extension length of 0.13 m.

**Figure 6:**
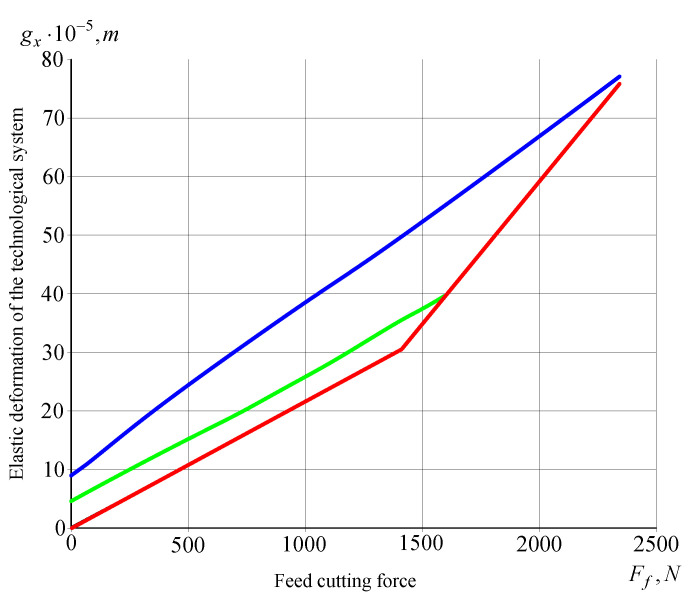
*X* Static characteristics for the *X* coordinate.

Due to the changes in the elastic system’s gain coefficients, during turning tests with blanks secured in the lathe centers, the values of elastic deformations $g_{y}$, even when a blank’s compliance was neglected, depended on the place where the cutting force was applied relative to the headstock and the tailstock. Accordingly, the values of coefficient $h_{yy}$ were established for specific processing conditions by the so-called “industrial method”, in which the values of elastic deformations in relation to the *Y* axis are assessed on the basis of the machining accuracy of the blank. Once the machining accuracy, i.e., value $g_{y}$, is known and the cutting force has been determined experimentally or analytically, one can determine coefficient $h_{yy}$. Due to the high rigidity of the blank relative to the *X* axis, the gain coefficient $h_{xx}$ of the elastic system mainly depended on the compliance of the headstock and the machine tool’s carriage assembly. The experimental static curve $g_{x}\left(F_{f}\right)$ is shown in Figure 6.

### 3.2. Investigations of the Time Characteristics of the Object under Interference and under Longitudinal Feed Control

The dynamic characteristics of the object were studied using active experiment methods. To obtain the time characteristics, the curves of the object’s output coordinates were recorded over the time when the cutting edge was entering the blank.

**Figure 7:**
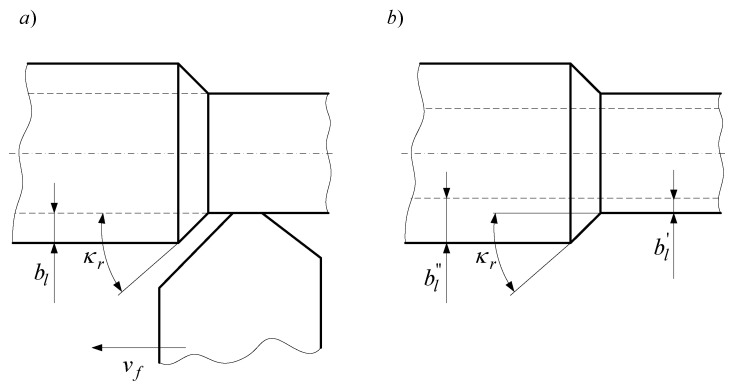
Sketches of the blanks used to determine the transition function: (**a**) under longitudinal feed control, (**b**) under interference in the form of change in allowance.

It should be noted that the process of the cutting edge cutting into the blank at constant values of longitudinal feed and rotational speed of the blank can be considered as a transient process for both the control parameter and the interference. At the same time, the two types of effect can be considered as step effects if the main cutting edge of the cutting tool is positioned in parallel to the machined surface and the thickness of the machined layer remained constant after the cutting edge has entered the blank. Based on these assumptions, workpieces with multiple step changes in the machining allowance were especially prepared, which made it possible to repeatedly measure the object’s unit step response during the turning of one workpiece. Transient processes are characterized by zero initial conditions. Transient characteristics of the object for a disruption at non-zero initial conditions were registered during turning tests with a step change in machining allowance (Figure 7).

During the experimental tests, the tangential component of the cutting force was adopted as the object’s output signal. It was measured using the dual-circuit strain gauge. Under the action of the tangential cutting force, the “movable” part of the strain gauge, in which the cutting edge was secured, moved in relation to the “stationary” part mounted on the carriage, as a result of the elastic deformations of the part with a reduced cross-section. Elastic displacement was measured using an inductive linear displacement sensor. As the results of the experiments show, the value of elastic displacement of the “movable” part of the strain gauge regarding the “stationary” part depended practically solely on the tangential cutting force, due to the high stiffness of the part with a cross-section reduced in the axial and radial directions.

To register the static characteristic of the strain gauge, a force acting in the same direction as the tangential cutting force was applied to the cutting edge secured in the strain gauge with a special jack. As a result of these experiments, the gain coefficient of the strain gauge was found, and it was established that the non-linearity of the gauge’s static characteristic did not exceed 2%. The dynamic characteristics of the strain gauge were obtained by registering the curves of the transient processes induced by an increase and a decrease in the load. The inertia of the strain gauge, as shown in the experiments, was an order of magnitude lower than the inertia of the object, which means the strain gauge could be regarded as a proportional element.

In order to accurately capture the beginning of the transient process at the moment when the cutting edge cut into the blank during computer registration, the cutting tool, electrically insulated from the tool holder, was supplied with voltage from a low voltage source, and the other pole was connected to the casing of the machine tool. The moment when the cutting edge of the cutter made contact with the workpiece was determined to be the closing of the electrical circuit and was recorded on the measurement card. The sampling period in most of the experiments was 1 ms.

**Figure 8:**
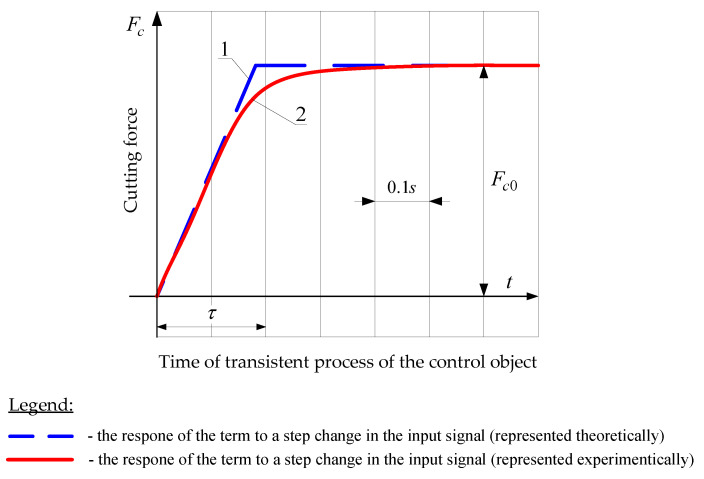
Experimental transient curve of the control object.

**Figure 9:**
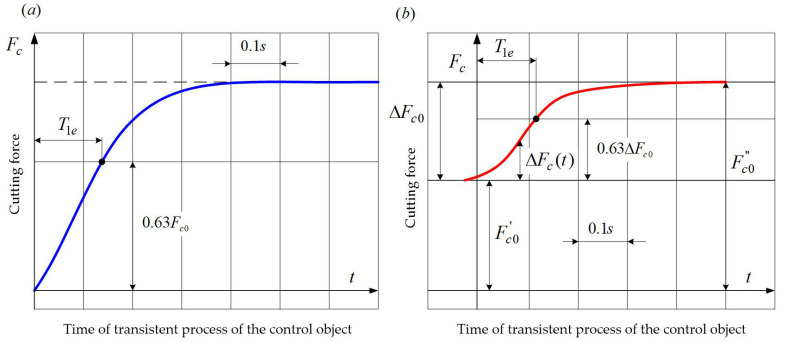
Experimental transient curves of the control object: (**a**) A transient curved obtained during the cutting process, (**b**) a second-order aperiodic link adopted as an approximate model.

For example, Figure 8 and Figure 9 show the curves of the transient processes obtained during the experimental tests of the dynamic system of the lathe.

The first curve (Figure 8) was obtained for the cutting process under the following conditions: stock material—*C45* steel (chemical composition: C: 042–05%; Mn: 05–08%; Si: 01–04%; S: max 0,04; Cr: max 0,3; Ni: max 0,3; Mo: max 0,1; Cu: max 0,3), cutter with an *S10* insert (geometry: γ = −5°, α = 6°, r_ε_ = 0.8 mm; chemical composition: WC—56%; TiC + TaC + NbC—35%; Co—9%), entering angle $\kappa_{r}=90^{{}^{\circ}}$, cutting speed *v_c_* = 90 m/min, depth of cut *a_p_* = 1 mm, longitudinal feed rate *v_f_* = 60 mm/min, thickness of the machined layer in steady state a = 0.2 mm, value of the tangential cutting force in steady state *F_c0_* = 350 N, blank revolution time *τ* = 0.2 s, blank diameter *d* = 30 mm.

The gain coefficients of the machining process were calculated (taking into account reference data): *m_x_* = 0.66 × 10^6^ N/m, *m_y_* = 0.14 × 10^6^ N/m. The compliance of the blank, as shown by the calculations, can be neglected in the case under consideration. The static characteristics (Figure 5 and Figure 6) were used to determine *h_xx_* = 1.5 × 10^−7^ m/N and *h_yy_* = 0.66 × 10^−7^ m/N. The value of coefficient *B* was calculated using formula (1):

(1) $$G_{v_{f}Fi}(s)=\frac{\Delta F_{i}(s)}{\Delta v_{f}(s)}=\frac{K_{1v_{f}Fi}G_{\tau }(s)}{s}=\frac{K_{1v_{f}Fi}}{s}(1-e^{-s\tau })$$

The approximate mathematical model can take the form of an integral term with a transition function (2):

(2) $$G_{o}(s)=\frac{\Delta Y_{o}(s)}{\Delta v_{f}(s)}=\frac{K_{o}}{s}\left(1-e^{-s\tau }\right)$$

The response of the term to a step change in the input signal is represented theoretically by a signal linearly increasing in time *τ* (curve 1 in Figure 8). The experimental curve of the output signal 2 is close enough to the theoretical curve; the maximum deviation is 12%.

Figure 9a shows a transient curve obtained during the cutting process performed under the following conditions: stock material—*C45* steel, cutter with an *S10* insert, *κ_r_* = 45°, *v_c_* = 96 m/min, *a_p_* = 1 mm, *v_f_* = 100 mm/min, a = 0.2 mm, value of the tangential cutting force in steady state *F_c0_* = 1450 N, *τ* = 0.12 s. The gain coefficients of the cutting process and the elastic system were determined, and the value of coefficient *B* was calculated (*B* = 1.2). Model (3), which takes the form of a second-order aperiodic link, should be adopted as an approximate model taking into account the value of parameter *B*,

(3) $$G_{v_{f}Fi}(s)=\frac{\Delta F_{i}(s)}{\Delta v_{f}(s)}=\frac{K_{v_{f}Fi}}{\left(T_{o1}s+1)(T_{o2}s+1\right)},$$

where:

(4) $$K_{v_{f}gi}=K_{1v_{f}gi}\tau ,\;K_{v_{f}Fi}=K_{1v_{f}Fi}\tau$$

(5) $$T_{o1,o2}=0.5\tau \left[0.5+B\pm \sqrt{{\left(0.5+B\right)}^{2}-\frac{1}{3}}\right].$$

The time constants determined using formula (5) are *T_o1_* = 0.2 s and *T_o2_* = 0.006 s. Given that the second time constant of the object is by an order smaller than the first one, one can assume, for further calculations, that *T_1p_* = *T_o1_*, and approximate the experimental curve with an exponential function; the time constant can then be set at *T_1e_* = 0.18 s, as the time after which the output signal reaches 0.63 of its steady state value. The estimation error of the computed time constant is:

(6) $$\delta =\frac{T_{1e}-T_{1p}}{T_{1e}}*100\%=-11\%.$$

The time curve of the object obtained during the machining of a blank with a step change in the depth of cut from *a*_*p*1_ = 1.5 mm to *a*_*p*2_ = 3 mm (i.e., a change in allowance Δ*a_p_* = 1.5 mm), is shown in Figure 9b. Stock material—*C45* steel, cutter with an *S10* insert, *κ_r_* = 45°, *v_c_* = 98 m/min, *v_f_* = 100 mm/min, *a* = 0.2 mm, *τ* = 0.075 s, the value of the tangential cutting force in steady state *F′*_*c*0_ = 620 N, *F″*_*c*0_ = 1240 N. According to the above-mentioned relationship, the mean calculated value of coefficient *B* = 0.9. Correspondingly, the time curve of the object was approximated by an exponential curve with the computed time constant *T*_1*p*_ = 0.106 s. The transient process was characterized by non-zero initial conditions. The experimental value of the time constant *T*_1*e*_ = 0.1 s was determined from the output signal curve. The estimation error for the time constant calculated using formula (6) was −6%.

Table 1 provides basic information about the experimental machining conditions, as well as the calculated and experimental values of the time constants of the transient process.

The experimental transient curves were approximated by formula,

(7) $$F_{c}(t)=F_{c0}\left[1-\exp(-t/T_{1e})\right]$$

where: *F_c0_*—steady state value of the output signal or its increment, *T_1e_*—equivalent time constant determined on the basis of the response curve as the time after which the output signal or its increment reach 0.63 of the steady state value.

**Figure 10:**
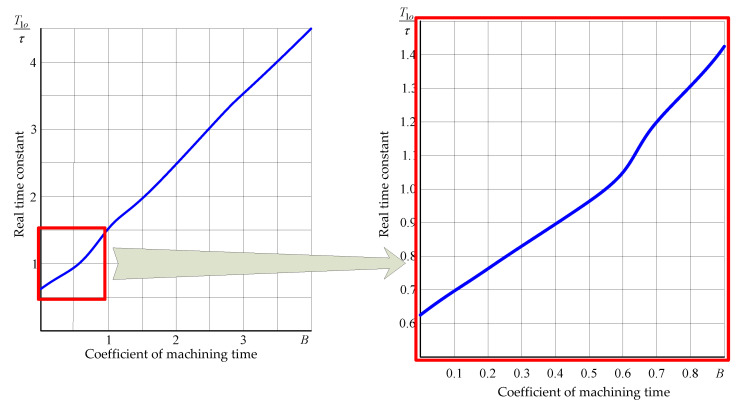
Graphs for calculating the values of the time constants *T*_1*o*_ of the control object.

The experimental values T*_1e_* were compared with the calculated ones *T_1p_*. The latter were defined as the time when the transient characteristic reached 0.63 of its steady-state value. The relative values of *T_1p_* depend on coefficient *B* and can be determined from the curves, shown in Figure 10.

The results reported above were obtained during the machining of *C45* steel blanks using a cutter with an *S10* insert at entering angles of 45° and 90°. The values of the time constants, shown in Table 1, were calculated as a means from three cutting curves obtained under the same conditions. The time constants were determined analytically using the values of the gain coefficients of the elastic system and the machining process determined during the experiments. The time constant calculation errors do not exceed 20%.

In this way, the results of the experimental studies of the control characteristics of the object (longitudinal feed rate and spindle speed) and the interference acting on it (a change in the machining allowance along the circumference of the workpiece) prove that the mathematical models, obtained by analytical identification, are satisfactory. Our study confirms the conclusion that the parameters of the control object can change over a wide range of values.

### 3.3. Study of the Frequency Characteristics of the Object for the Feed Channel

Simultaneously with the time characteristics, we investigated the frequency characteristics of the machine tool system while controlling the longitudinal feed rate.

Tests of the frequency characteristics of the object in the feed channel were carried out on the same lathe, equipped with a longitudinal feed sensor and a two-component strain gauge. The signal, which included harmonic components and a constant, was fed to the input of the *CNC* machine feed generator, from whose output it travelled via an interpolator and commutator to the input of the longitudinal feed drive. The frequency characteristics were registered while turning *C45* steel blanks at spindle speeds of 90, 710 and 1400 rpm using a cutter with an *S10* insert. To assess the adequacy of the object’s model, the experimental and calculated frequency characteristics were compared. To calculate the amplitude and phase characteristics, the relationships of the baseline model presented in the form of Equation (8) were used,

(8) $$A\left(\omega \right)=K_{0}\frac{1}{\omega \sqrt{C_{1}}},\;\;\varphi \left(\omega \right)=-\frac{\pi }{2}-arc\;ctg\left(\frac{ctg\left(\omega \tau /2\right)}{1+2B}\right)$$

where: $C_{1}=\;B^{2}+\;B+0.25+0.25\;ctg^{2}\left(\omega \tau /2\right).$

**Figure 11:**
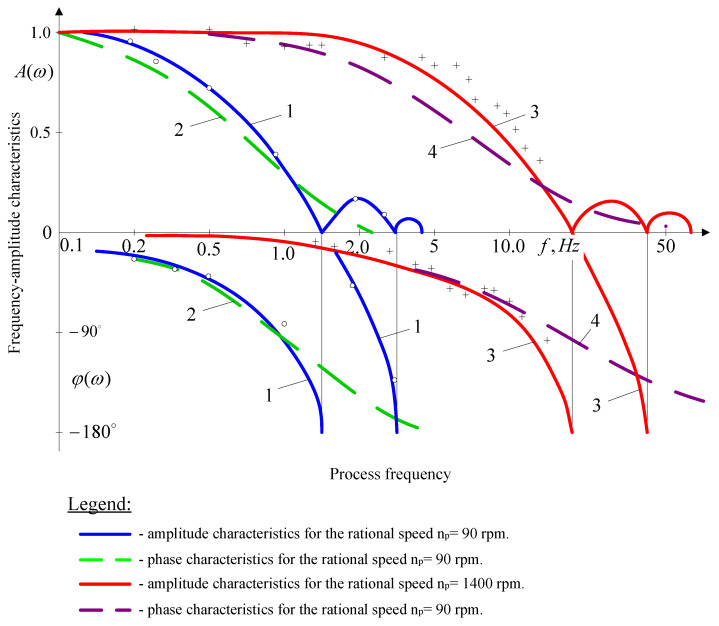
Frequency (amplitude and phase) characteristics of the control object under longitudinal feed control.

Figure 11 shows the amplitude and phase frequency characteristics obtained in relative units of a semi-logarithmic scale at spindle speeds of 90 rpm (curve 1) and 1400 rpm (curve 3) and the amplitude and phase frequency characteristics (curves 2 and 4) corresponding to the approximate model (9) in the form of an aperiodic second-order link:

(9) $$G_{v_{f}gi}(s)=\frac{\Delta g_{i}(s)}{\Delta v_{f}(s)}=\frac{K_{v_{f}gi}}{\left(T_{o1}s+1)(T_{o2}s+1\right)}.$$

Experimental data for the rotational speed *np* = 90 rpm (cutter with an *S10* insert, *κ_r_* = 55° blank diameter *d*= 58 mm, depth of cut *a_p_* = 2.5 mm, coefficient *B* = 0.22) are plotted as circles and those for the rotational speed *n_p_* = 1400 rpm (cutter with an *S10* insert, *κ_r_* = 55°, blank diameter *d* = 30 mm, depth of cut *a_p_* = 2.5 mm, coefficient *B* = 0.25) are plotted as crosses (Figure 11).

The amplitude and phase curves of the object have a number of characteristic points: for the critical frequency values, the amplitude curve takes zero values, and the phase curve has discontinuities. For *n* = 90 rpm, the critical values of the cyclic frequency are: *f_k_* = k/π (k = 1, 2, 3, …) *f*_*k*1_ = 1.5 Hz, *f*_*k*2_ = 3 Hz, *f*_*k*3_ = 4.5 Hz. As Figure 10 shows, the experimental data for longitudinal turning of structural steel blanks are in good agreement with the data calculated using the baseline-model equation (8). For approximate models with the transition function (10), the agreement between the experimental and calculated data for frequencies smaller than the first critical value, which are the most important from the point of view of the synthesis of an automatic control system, is satisfactory for practical calculations:

(10) $$G_{v_{f}gi}(s)=\frac{\Delta g_{i}(s)}{\Delta v_{f}(s)}=\frac{K_{v_{f}gi}}{\left(T_{o1}s+1)(T_{o2}s+1\right)},$$

The results demonstrate that the theoretical curves, calculated for the basic/baseline model of the object, are in good agreement with the experimental data. In the case of approximate models in the form of a second order aperiodic link, the analytical and experimental results are sufficiently accurate for the range of frequencies lower than the first critical frequency.

In this study, a number of measurement series with data from the active experiment were obtained, which were used in the study of models of the turning process with real-time parameter estimation.

## 4. Conclusions

The provided research and obtained results provide the possibility of drawing the following conclusions.

1. The experimental tests assessed the characteristics of the control object’s control parameters of longitudinal feed rate and spindle speed, as well as the interference parameter in the form of a change in the allowance around the circumference of the workpiece. The results shows that the models obtained by analytical identification methods exhibit satisfactory usefulness. The tests demonstrate that the parameters of the control object can change over a wide range.
2. We conducted an analysis of the frequency characteristics of the models of dynamic systems of machine tools for the input signals of longitudinal feed rate, change in the hardness of the stock material and change in the allowance along the axis of the workpiece. We established that the amplitude frequency curves and the phase frequency curves of the models represented periodic frequency functions with a period that was a multiple of 1/π.
3. The frequency characteristics of the object for the feed rate channel demonstrate that the theoretical curves, calculated for the baseline model of the object, fit the experimental data well. In the case of approximate models in the form of a second order aperiodic link, the analytical and experimental results are sufficiently accurate for the range of frequencies lower than the first critical frequency.
4. The analysis of the frequency and time curves shows that the gain coefficients and time constants of the approximate models change primarily due to changes in the rotational speed of the workpiece and the complex coefficient characterizing the ratio of the rigidity of the elastic system and cutting coefficients. In the case of universal lathes and grinding machines, model parameters may change dozens of times due to changes in operating conditions. The time constraints for the machining process for the wide range of machining parameters change from 0 to 26%
5. The results of the present experimental study of the frequency and time characteristics of the object for various control and interference parameters confirm the usefulness of the models formulated on the basis of analytical identification methods. They can be used for developing the control system that allows to reduce both the variation of the machining force and elastic deformations. As a consequence, the accuracy of the machined parts can be increased.

## Figures and Tables

**Table 1:** Cutting parameters and experimental and computed time constants [32].

Nr	*τ*, s	*κ_r_*	*v_c_* m/s	*a_p_*, mm	*a*	*F*_*c*0_, N	*T*_1*e*_, s	*T*_1*p*_, s	*δ*, %
1	0.48	45	0.8	2.0	0.2	900	0.59	0.55	7
2	0.48	45	0.8	3.0	0.2	1380	0.7	0.625	11
3	0.375	45	0.85	1.0	0.2	480	0.3	0.3	0
4	0.375	45	0.85	2.0	0.25	1140	0.46	0.43	7
5	0.375	45	0.85	3.0	0.2	1400	0.55	0.48	13
6	0.24	45	1.3	1.5	0.25	855	0.23	0.215	7
7	0.24	45	1.3	2.0	0.25	1150	0.34	0.28	18
8	0.24	45	1.3	3.0	0.2	1370	0.36	0.31	14
9	0.12	45	1.6	1.5	0.2	730	0.12	0.13	−8
10	0.12	45	1.6	3.0	0.2	1470	0.18	0.161	11
11	0.095	45	1.65	1.0	0.2	475	0.08	0.076	5
12	0.095	45	1.65	3.0	0.2	1475	0.15	0.13	13
13	0.095	45	1.65	5.0	0.1	1180	0.2	0.19	5
14	0.075	45	1.67	1.0	0.2	470	0.06	0.064	−7
15	0.075	45	1.67	2.0	0.2	981	0.11	0.09	18
16	0.075	45	1.67	3.0	0.2	1430	0.12	0.105	5
17	0.048	45	1.69	1.0	0.2	390	0.04	0.04	0
18	0.048	45	1.69	3.0	0.2	795	0.072	0.065	10
19	0.48	90	0.9	1.0	0.25	610	0.3	0.38	−26
20	0.48	90	0.9	2.0	0.25	1200	0.42	0.43	−2
21	0.19	90	1.33	2.0	0.25	1100	0.18	0.17	6
22	0.19	90	1.33	5.0	0.1	1270	0.32	0.37	−16
23	0.12	90	1.7	1.0	0.25	590	0.088	0.096	−9
24	0.12	90	1.7	3.0	0.25	1770	0.16	0.14	12
25	0.095	90	1.65	1.0	0.25	580	0.07	0.076	7
26	0.095	90	1.65	5.0	0.1	1200	0.16	0.18	13

## Data Availability

The data presented in this study are available on request from the corresponding author.

## Nomenclature

*F_c_*	*cutting force*
*F_p_*	*passive force*
*F_f_*	*reed force*
*h_ij_*	coefficients that describe the compliance of a technological system,
*g_y_*(*Fp*)	characteristic of the elastic system consisting of the headstock and the carriage,
*g*	elastic deformation of the technological system,
*F*_p_	radial cutting force,
*h_xx_*	amplification coefficient of the elastic system,
*κ_r_*	entering angle,
*v_c_*	cutting speed,
*a_p_*	depth of cut
*v_f_*	longitudinal feed rate,
*a*	thickness of the machined layer in steady state,
γ	insert angle of attack,
α	insert clearance angle,
r_ε_	corner radius of the insert,
*F*_*c*0_	value of the tangential cutting force in steady state,
*τ*	blank revolution time,
*f*	frequency,
*d*	blank diameter,
*m_x_, m_y_*	the gain coefficients of the machining process,
*T*_*o*1_, *T*_*o*2_	the time constants,
*S*	Laplace’s operator,
B	coefficient of machining process (being the combination of products of amplification coefficients of machining process),
*T*_1*e*_	equivalent time constant determined on the basis of the response curve,
*T*_1*p*_	calculated time constant determined on the basis of the response curve,
*T*_l0_	real time constant,
*f*	frequency
*f_k_*	the critical value of the cyclic frequency,
*I*	amperage
*V*	voltage
*PC*	personal computer,
*CNC*	computer numerical control.
